# Management of mad honey intoxication with suspected anaphylaxis in Nepal: a case report

**DOI:** 10.1097/MS9.0000000000000800

**Published:** 2023-05-10

**Authors:** Ayush Anand, Nabin Adhikari, Ashwini Gupta, Rajesh Ranjan, Arun Gautam, Urza Bhattarai, Bhupendra Shah

**Affiliations:** a BP Koirala Institute of Health Sciences, Dharan, Nepal; b Department of Internal Medicine, BP Koirala Institute of Health Sciences, Dharan, Nepal

**Keywords:** anaphylaxis, case report, intoxication, mad honey, Nepal

## Abstract

Consumption of mad honey can lead to intoxication. The exact incidence of mad honey-induced intoxication is unknown. Typically, the patients present with dizziness, nausea, syncope, and sinus bradycardia.

**Case presentation:**

The authors reported the case of a middle-aged male patient who presented with blurring of vision, passage of loose stools, vomiting, and profuse sweating after ingestion of honey. He also had a history of loss of consciousness. On presentation, he was hypotensive and tachypneic with cold, clammy extremities. His ECG showed sinus bradycardia. The authors made a diagnosis of mad honey intoxication with suspected anaphylaxis. The authors treated him with intravenous normal saline, epinephrine, and atropine. He again developed hypotension and bradycardia in a few hours, for which hydrocortisone was administered, following which his heart rate was normalized in 2 h. Overall, the recovery time in our patient was 8 h. The patient was counseled to avoid consuming mad honey and did well on his monthly follow-up.

**Discussion:**

Our patient had signs and symptoms suggesting intoxication following ingestion of mad honey with suspicion of anaphylaxis. Similar to other reported cases, the patient had sinus bradycardia and hypotension. Epinephrine and atropine were administered to treat hypotension and bradycardia, respectively. Also, refractory hypotension was managed by intravenous hydrocortisone. Usually, atropine and saline infusion are sufficient to manage these cases, and simultaneous use of epinephrine and atropine should be avoided unless indicated.

**Conclusion:**

Our case highlighted the approach to diagnosing and treating mad honey intoxication with suspected anaphylaxis.

## Introduction

Highlights

- Mad honey intoxication can present with features of cardiovascular and gastrointestinal involvement.
- Anaphylaxis can sometimes present with early bradycardia and hypotension.
- Atropine and saline infusion are sufficient for the management of mad honey intoxication.
- Clinicians should avoid the simultaneous use of epinephrine and atropine unless indicated.
- In mad honey intoxication cases, anaphylaxis should be considered a differential diagnosis.

Mad honey is produced by honeybees from the nectar of Rhododendron plant species, mainly found in Turkey, Japan, Europe, parts of North America, and the hilly regions of Nepal^1^. The mad honey intoxication is primarily due to the persistent activation of sodium channels by grayanotoxins in the mad honey^2^. Usually, the patients are males, presenting with dizziness, nausea, syncope, and ECG findings of sinus bradycardia and atrioventricular block^3^. And, the management involves close monitoring of vitals and administering atropine and intravenous fluids^3^. So far, no mortality has been reported, and patients can be discharged within 24 h of recovery^3^. Herein, we present the case of successful management of mad honey intoxication with suspected anaphylaxis in a 41-year-old male from Nepal.

## Case presentation

### History

A 40-year-old Nepali male presented to the emergency department (ED) of a tertiary care hospital with blurring of vision, passage of loose stools, vomiting, and profuse sweating following ingestion of honey bought from the Taplejung district of Nepal. The patient developed blurring of vision ~15 min following the consumption of three tablespoons of wild honey. The patient had two episodes of loose stool followed by vomiting, after which he lost consciousness for half an hour. After he gained consciousness, he had another episode of vomiting, following which he was rushed to the ED. There was no history of an allergic/anaphylactic reaction following the consumption of honey, angioedema, drugs, exposure to hymenoptera, or pollen allergy. There was no history of allergy, bronchial asthma, or any documented allergies in family members.

### Physical examination

On physical examination, the patient was conscious and oriented to time, place, and person but had a feeble pulse with a cold and clammy periphery. He had bradycardia [a pulse of 38 beats per minute (bpm)], tachypnea (respiratory rate of 24 breaths per minute), hypotension (systolic blood pressure of 60 mm Hg and a diastolic blood pressure that was not recordable), and normoxia (oxygen saturation of 96%). He was alert and had a clear airway during his presentation to the ED. A detailed systemic examination was not done due to need for urgent intervention in an emergency setting.

### Investigations

**Figure 1:**
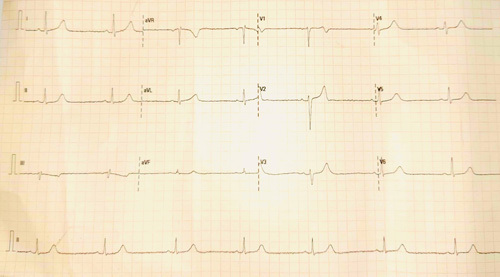
Electrocardiogram on initial presentation showing sinus bradycardia.

An ECG showed sinus bradycardia with a heart rate of 42 bpm without any conduction defects (Fig. 1). Initial laboratory investigations (Table 1) revealed elevated creatinine kinase-MB.

**Table 1:** Laboratory investigations of the patient

Test	Result	References
Hemoglobin (mg/dl)	15.4	11–16
TLC (cells/mm^3^)	7840	4000–11 000
DLC (%)	N54 L40	N40-75 L20-45
Platelets (cells/mm^3^)	193 000	150 000–400 000
MCH (pg)	32.4	27–34
MCHC (g/dl)	35.3	32–36
MCV (fl)	91.7	80–96
PT (seconds)	16	12–16
INR	1.2	
RBS (mg/dl)	105	80–140
Serum Urea (mg/dl)	34.7	10–50
Serum Creatinine (mg/dl)	1.0	0.6–1.3
Serum Sodium (meq/l)	147	135–150
Serum potassium (meq/l)	4.2	3.5–5.1
Liver function test		
Total protein (g/dl)	7.1	6.0–8.3
Albumin (g/dl)	4.6	3.5–5.0
Total bilirubin (mg/dl)	1.3	0.2–1.2
Conjugated bilirubin (mg/dl)	0.3	<0.3
ALT (U/L)	58.1	09–43
AST (U/L)	41.5	10–35
ALP (U/L)	95.8	35–130
Cardiac enzymes		
CK-MB (IU/L)	32.8	5–25
CK NAC Accent (IU/L)	99.2	24–195

ALP, alkaline phosphatase; ALT, alanine transaminase; AST, aspartate transaminase; CK NAC Accent, Creatine kinase N-acetylcysteine Accent; CK-MB, Creatine kinase-MB; DLC, differential leukocyte count; Hb, hemoglobin; INR, international normalized ratio; MCH, mean corpuscular hemoglobin; MCHC, mean corpuscular hemoglobin concentration; MCV, mean corpuscular volume; PT, prothrombin time; RBC, red blood cell; TLC, total leukocyte count; WBC, White blood cells.

### Diagnosis, intervention, and follow-up

Based on the clinical evaluation, the team comprising an emergency medicine physician, an internist, a cardiologist, and a neurologist diagnosed mad honey intoxication with suspected anaphylaxis. The patient was immediately resuscitated with intravenous 0.9% normal saline, and intramuscular epinephrine 0.5 mg stat was used to manage anaphylaxis. Also, intravenous atropine 1 mg was added to manage sinus bradycardia. The patient’s blood pressure improved to 180/120 mm Hg over 10 min, and his heart rate improved to 102 bpm. However, over 5 h, his blood pressure decreased to 90/60 mm Hg and heart rate to 42 bpm. Injection hydrocortisone 100 mg intravenous was administered, which improved the blood pressure to 140/80 mm Hg over 10 min, and the heart rate gradually improved to 62 bpm over 2 h. Following this, the patient was shifted to the medicine ward and kept under close observation with maintenance fluid. The patient was discharged the next day without complications during his stay in the medicine ward. On discharge, the patient was counseled to avoid taking mad honey in the future. On follow-up, after one month, the patient is doing well. We have reported our work in line with Surgical CAse REport (SCARE) 2020 criteria^4^.

## Discussion

A systematic review by Silici and Atayoglu^3^ revealed that approximately three-fourths of the patients with mad honey intoxication were males, most commonly in the middle-aged group. Approximately half of the patients had dizziness and bradycardia^3^. Most patients had heart rates between 30 and 60 bpm, with a mean heart rate of 47.11±15.87^3^. Other complaints were vomiting in 35.50%, blurring of vision in 20.42%, hypotension in 19.75%, impaired consciousness in 11.67%, sweating in 11.33%, and only 0.67% had diarrhea^3^. Also, the most commonly reported ECG finding was sinus bradycardia in 79.58% of patients^3^. Case reports from Nepal also reported hypotension and bradycardia following mad honey ingestion^5–8^. They reported vomiting, dizziness, weakness, and burning and tingling sensations over the whole body^5–8^. Similarly, our patient was a middle-aged male with a heart rate of 32 bpm accompanied by blurring of vision, vomiting, hypotension, loss of consciousness, profuse sweating, and diarrhea. In patients presenting with bradycardia, it is crucial to consider mad honey-induced anaphylaxis and vasovagal syncope as differentials^9,10^. Anaphylaxis usually presents with urticaria, wheezing, flushing, and tachycardia; bradycardia is usually a late manifestation^9,11–15^. Sometimes, bradycardia may be reported early in anaphylaxis, possibly due to histamine-mediated coronary vasospasm^16–18^. Hence, we diagnosed mad honey intoxication with anaphylaxis^3^. The majority of the patients with mad honey-induced intoxication reported consumption of 1 to 5 tablespoons of mad honey^3^. In our case, the symptoms developed after consuming three tablespoons of mad honey. Usually, supportive management with saline infusion and atropine is sufficient to manage these cases^3,8^. However, we managed with early administration of epinephrine, as anaphylaxis was suspected^13,15,19^. Also, our patient had bradycardia with a heart rate of less than 50 bpm. Based on American Heart Association guidelines, intravenous atropine was added to manage symptomatic bradycardia^19,20^. This led to the effective management of hypotension and bradycardia in the patient. However, the patient again developed hypotension and bradycardia after a few hours. We used intravenous hydrocortisone as it can significantly increase systolic and diastolic blood pressure and decreases the need for inotropic support, particularly in refractory hypotension cases^21–25^. After intervention with hydrocortisone, the blood pressure improved, and the heart rate was normalized in 2 h. The recovery time in mad honey-induced intoxication cases ranges from 2 to 73 h ^3^. In our patient, the recovery time was ~8 h. Following this, we kept the patient on maintenance fluids and under close observation. The patient remained hemodynamically stable and was discharged the following day. Though our case is unique, there are certain limitations. There was a sudden rise in blood pressure following the simultaneous use of atropine and adrenaline. This might have been potentially dangerous. However, due to suspected anaphylaxis with mad honey intoxication, the use of epinephrine and atropine was indicated. In cases with anaphylaxis, continuous adrenaline infusion following initial stabilization can help reduce the repeated use of epinephrine^19^. However, due to the initial sudden rise in blood pressure, we were cautious regarding epinephrine use and decided to closely monitor the patient and intervene if necessary.

## Conclusion

Our case highlighted the diagnostic and management approach in patients with mad honey intoxication with suspected anaphylaxis. There is a need for a detailed history with an emphasis on demographics to identify the offending agent and a thorough clinical evaluation to assess the signs for the diagnosis of anaphylaxis. Though mortality is rare in these cases, prompt management with saline infusion and atropine is recommended. Simultaneous use of adrenaline and atropine might lead to rapid blood pressure elevation and adverse consequences. However, for the management of anaphylaxis, epinephrine is indicated. In addition, these patients require close monitoring under the supervision of emergency medicine/critical care physicians.

## Ethical approval

Ethical approval was not required for this case report.

## Consent

Written informed consent was obtained from the patient for publication of this case report and accompanying images. A copy of the written consent is available for review by the Editor-in-Chief of this journal on request.

## Sources of funding

The authors did not receive any funding for this manuscript.

## Author contribution

A.A.: conceptualization, investigation, project administration, supervision, writing-original draft and writing-review and editing; N.B. and A.G.: conceptualization, investigation, project administration, writing-original draft and writing-review and editing; R.R., A.G., and U.B.: writing-original draft and writing-review and editing; B.S.: project administration, supervision, writing-original draft and writing-review and editing. All authors approved the final version of the manuscript and are accountable for all aspects of the work.

## Conflicts of interest disclosure

The authors have no conflict of interest to declare.

## Research registration unique identifying number (UIN)

1. Name of the registry: NA.
2. Unique Identifying number or registration ID: NA.
3. Hyperlink to your specific registration (must be publicly accessible and will be checked): NA.

## Guarantor

Bhupendra Shah is the guarantor.

## Provenance and peer review

Not commissioned, externally peer-reviewed.

## Patient Perspective

I am happy with the care provided at the hospital, and my condition will help other healthcare professionals to learn from my condition. Also, I want the general public to be cautious about this and seek early medical attention.
